# Intense chorus waves are the cause of flux-limiting in the heart of the outer radiation belt

**DOI:** 10.1038/s41598-022-26189-9

**Published:** 2022-12-15

**Authors:** S. Chakraborty, I. R. Mann, C. E. J. Watt, I. J. Rae, L. Olifer, L. G. Ozeke, J. K. Sandhu, B. H. Mauk, H. Spence

**Affiliations:** 1 Department of Mathematics, Physics and Electrical Engineering, Northumbria University, Newcastle upon Tyne, UK; 2 Department of Physics, University of Alberta, Edmonton, AB Canada; 3 Applied Physics Laboratory, Johns Hopkins University, Laurel, MD USA; 4 Institute for the Study of Earth, Oceans, and Space, University of New Hampshire, Durham, NH USA

**Keywords:** Magnetospheric physics, Space physics

## Abstract

Chorus waves play a key role in outer Van Allen electron belt dynamics through cyclotron resonance. Here, we use Van Allen Probes data to reveal a new and distinct population of intense chorus waves excited in the heart of the radiation belt during the main phase of geomagnetic storms. The power of the waves is typically ~ 2–3 orders of magnitude greater than pre-storm levels, and are generated when fluxes of ~ 10–100 keV electrons approach or exceed the Kennel–Petschek limit. These intense chorus waves rapidly scatter electrons into the loss cone, capping the electron flux to a value close to the limit predicted by Kennel and Petschek over 50 years ago. Our results are crucial for understanding the limits to radiation belt fluxes, with accurate models likely requiring the inclusion of this chorus wave-driven flux-limiting process, that is independent of the acceleration mechanism or source responsible for enhancing the flux.

## Introduction

Understanding the processes that are responsible for the observed complex dynamics of the outer zone electron Van Allen belts during geomagnetic storms remains an active topic of research. The flux of relativistic electrons trapped in the Earth’s Van Allen radiation belts can vary by several orders of magnitude in response to solar wind forcing (e.g.,^1^), and a number of wave-particle interactions have been proposed as contributing to the observed dynamics. For example, chorus waves are responsible for local acceleration (e.g., see^2–11^), longer period ultra-low frequency (ULF) waves are responsible for particle acceleration as a result of inward radial diffusion (e.g., see^12–21^), in addition to other wave-particle interactions such as electromagnetic ion-cyclotron (EMIC) waves that are responsible for loss of radiation belt electrons (e.g., see^22,23^), manmade VLF transmitter waves (e.g.,^24^) and plasmaspheric hiss (e.g.,^25^) are also thought to be able to scatter the electrons into the loss cone and lead to the corresponding evolution of the electron flux. In this paper, we examine the chorus wave activity that accompanies a flux-limiting process in the inner magnetosphere that occurs during geomagnetic storms, and demonstrate that the theoretical predictions of Kennel and Petschek^26^ accurately depict the behaviour of waves and electron fluxes in the outer Van Allen belt.

Recent work associated with the capping of ~ 10–100 keV electron flux by Olifer et al.^27^ has revisited the dynamics of the energetic electron population in the outer electron radiation belt, revealing evidence for an energy-dependent limit to the electron flux in the belts (see also^28^, and references therein). Olifer et al.^27^ analysed 70 geomagnetic storms during the period of operation of the NASA Van Allen Probes^29,30^ from $2012 - 2019$. As shown by Olifer et al.^27^, during a geomagnetic storm, the flux of lower energy electrons ($\sim < 700$ keV) in the outer radiation belt ($4< L^* < 6$) quickly reaches a maximum and this flux maximum is the same from storm to storm. Olifer et al.^27^ further demonstrated that the lower energy electrons at energies $\sim 10$s keV reach a flux cap before electrons at higher energies. The behaviour of the flux hints at the flux-limiting theory of Kennel and Petschek^26^, but without appropriate wave data, the interpretation was not fully confirmed.

Kennel and Petschek^26^ proposed that electron fluxes with energies of tens to hundreds of keV could become self-limited to a maximum level through the action of whistler-mode waves (see also e.g.,^28^). In the low-density regions of the Earth’s magnetosphere that coincide with the outer radiation belt, these waves are commonly known as whistler-mode chorus. In the Kennel–Petschek paradigm, once electron flux levels reach a theoretical limit, self-generated intense chorus waves lead to rapid scattering of electrons into the atmosphere to prevent any further increases in flux, and to return the flux to values close to the theoretical limit. Although the flux value at which this process is triggered does not represent an overall upper level for the short term electron flux, it does represent the asymptotic limit to which the flux returns after the action of the Kennel–Petschek process. For simplicity of terminology, and to be consistent with prior literature, throughout the rest of the paper, we will refer to this level of electron flux as the “KP limit”.

In a Kennel–Petschek scenario there is a quasi-steady balance between an external source of electrons in the energy range ~ 10–100 keV that leads to strongly-driven unstable whistler-mode waves, rapid scattering of electrons in pitch-angle due to the presence of this intense whistler-mode chorus wave population, and the loss of the “excess” electrons into the upper atmosphere once they are scattered into the loss cone. In addition, some form of electron temperature anisotropy is required to drive the waves unstable, but the theorised self-limiting process is independent of its form. Anisotropy could be due to temperature differences in directions parallel and perpendicular to the field, as frequently found in Earth’s magnetosphere (e.g.,^31^), or simply due to the constant presence of the atmospheric loss-cone^26^. Once the self-limiting process has begun, there should be a clear relationship between the amount of electron flux above the KP limit, and the production of intense chorus waves. While Olifer et al.^27^ demonstrated that fluxes were capped at the KP limit, they did not examine the nature of the simultaneous chorus wave activity. Here we present evidence from a large number of geomagnetic storms demonstrating that the electron flux is limited through the generation of intense chorus waves in the heart of the Van Allen belts, exactly as predicted by theoretical analysis over fifty years ago.

Our results show that when flux of the lower energy source electron population (~10 s of keV) reaches close to or exceeds the KP limited flux, the most intense chorus waves are generated in the outer radiation belt. We further demonstrate how these intense waves represent a distinct and new population, whose occurrence is limited in time around storm main phase. The occurrence distribution of this distinct, intense wave population shows that extreme chorus wave power is dominant during main phase of geomagnetic storms. The results of this analysis are crucial for establishing the physical process through which fluxes are limited in the Van Allen belts, and also how the excess electron flux is lost to the upper atmosphere.

## Results

In this section, we present the relationship between the fluxes of electrons with energies of tens of keV and chorus wave power in the outer radiation belt as observed by Van Allen Probe-A. We used observations of the magnetic field wave spectra provided by the EMFISIS instrument on board the Van Allen Probe-A spacecraft^32^. To calculate the chorus wave power (in units of nT^2^), we integrated observations from the EMFISIS instrument from 0.1 to 0.8 of the electron equatorial gyro-frequency. Therefore, by chorus wave power, $P_{ch}$, we mean the integrated chorus magnetic field wave power. We first begin with presenting a typical example of simultaneous observations of very intense chorus waves and high fluxes of tens of keV electrons observed for a single geomagnetic storm before proceeding with the statistical analyses for all the storms using a superposed epoch approach. The methodology used to obtain the results presented in this section are described in detail in Section “Methods”.

**Figure 1:**
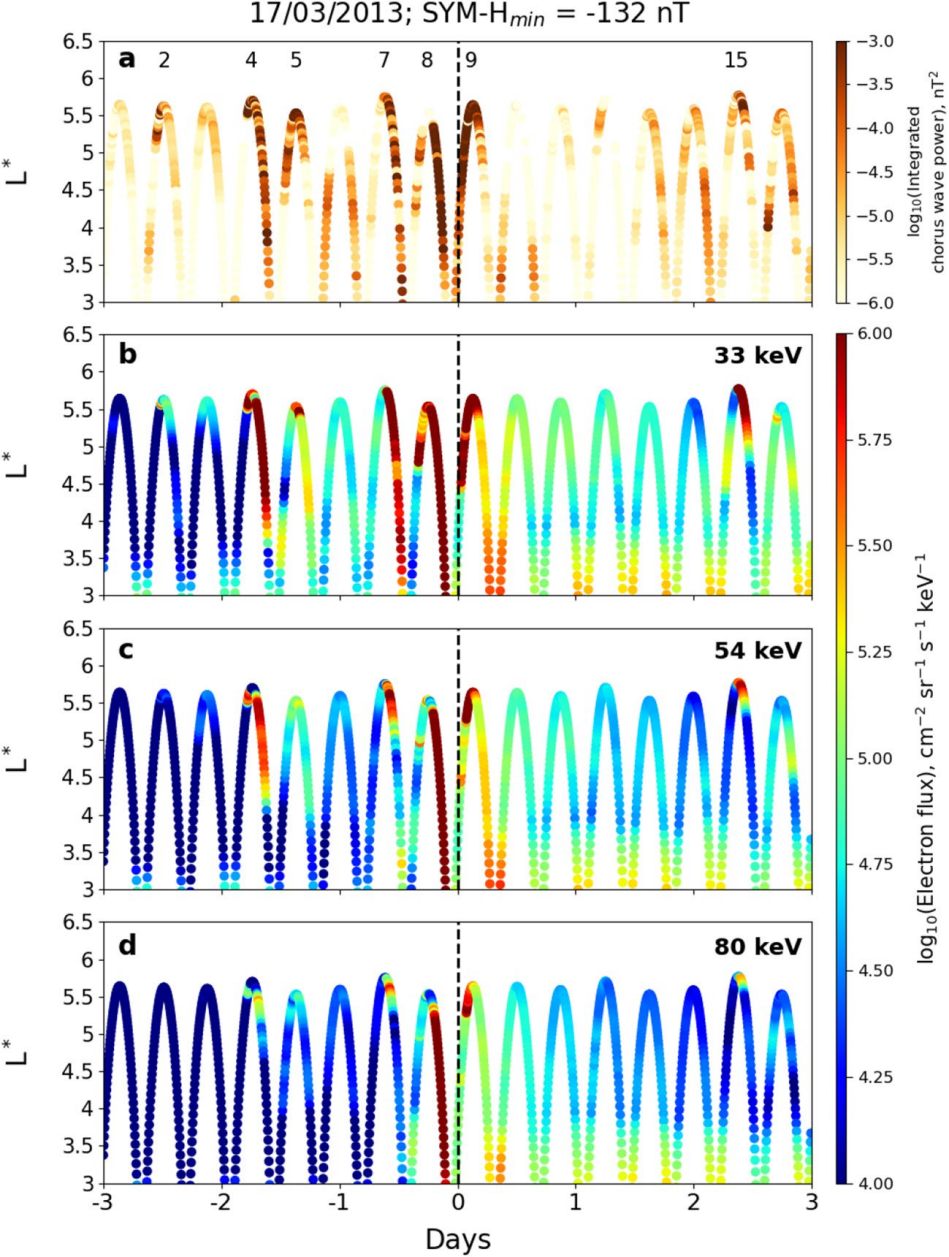
(**a**) Integrated chorus wave power, $P_{ch}$, and electron fluxes in three energy channels: (**b**) 33 keV, (**c**) 54 keV, and (**d**) 80 keV in logarithmic scale as a function of $L^*$ and time as observed by Van Allen Probe-A during the geomagnetic storm on March 17, 2013. The observations are made over a period of 6 days starting from 3 days before minimum SYM-H (day 0, marked by vertical dotted line in each panel) to 3 days after minimum SYM-H. The colorbars at the right denote the corresponding integrated chorus wave power (panel **a**) and electron fluxes in logarithmic scale (panels **b**–**d**). In panel (**a**), the orbit numbers in which intense chorus waves (wave power typically $> 10^{-4}$ nT^2^) are observed are also indicated.

Figure 1 shows (a) the integrated chorus wave power $P_{ch}$ (in nT^2^) for the frequency range $0.1< f < 0.8f_{ce}$, where $f_{ce}$ is the equatorial electron gyrofrequency; and differential electron fluxes (in cm$^{-2}$ sr$^{-1}$ s$^{-1}$ keV$^{-1}$) for three energy channels: (b) 33 keV, (c) 54 keV and (d) 80 keV. Observations are presented on a logarithmic scale, and are obtained by the Van Allen Probe-A spacecraft during the 2013 St. Patrick’s day geomagnetic storm. The time of minimum SYM-H (− 132 nT; 20:30 UT on March 17, 2013) is taken as epoch day 0. The orbits in which intense chorus waves are observed are indicated in panel (a) using colour to indicate the wave power. Interestingly, it is during the same orbits and the same L$^*$ ranges within those orbits that very high electron fluxes are also observed (panels b, c, d). Such simultaneous observations of very intense chorus waves and electron fluxes are obtained consistently throughout all the 70 geomagnetic storms in the Van Allen Probe era ($2012 - 2019$) studied in this paper. As mentioned in Section “Introduction”, it is also these same 70 geomagnetic storms during which Olifer et al.^27^ showed statistically that the lower energy fluxes are largely capped by the KP limit. The strong spatio-temporal correlation between intense chorus waves and electron fluxes in the individual case study shown in Fig. 1, directly suggests that large values of tens of keV electron flux may act as the causative agent for generating very intense chorus waves in the outer radiation belt.

We now examine the statistical relationship between the magnitude of the energetic electron flux and the presence of intense chorus waves. In particular, we monitor the difference between the observed flux and the approximate KP limit over three different energy channels in the 10–100 keV range. For this study, we considered 70 geomagnetic storms identified in the period $2012 - 2019$. The statistical analyses include superposed epoch analysis, with zero epoch defined as the time of minimum SYM-H. We again use the integrated chorus wave power $P_{ch}$ for $0.1< f < 0.8f_{ce}$, and the ratio between the observed flux and the KP limited flux (on a log scale; see Section 4 for details) to perform the statistical analyses. Further, here we present results from events observed by Van Allen Probe-A within the MLT range $0 - 12$ MLT. We restrict our analysis to this morning local time sector because past studies have shown that time-averaged mean-amplitude chorus waves have higher intensity in the local time sector $0 - 12$ MLT (see e.g.,^33–38^). A comparison of the variation of integrated chorus wave power and electron fluxes between $0 - 12$ MLT and $12 - 24$ MLT is provided in the supplementary material (Fig. S1).

**Figure 2:**
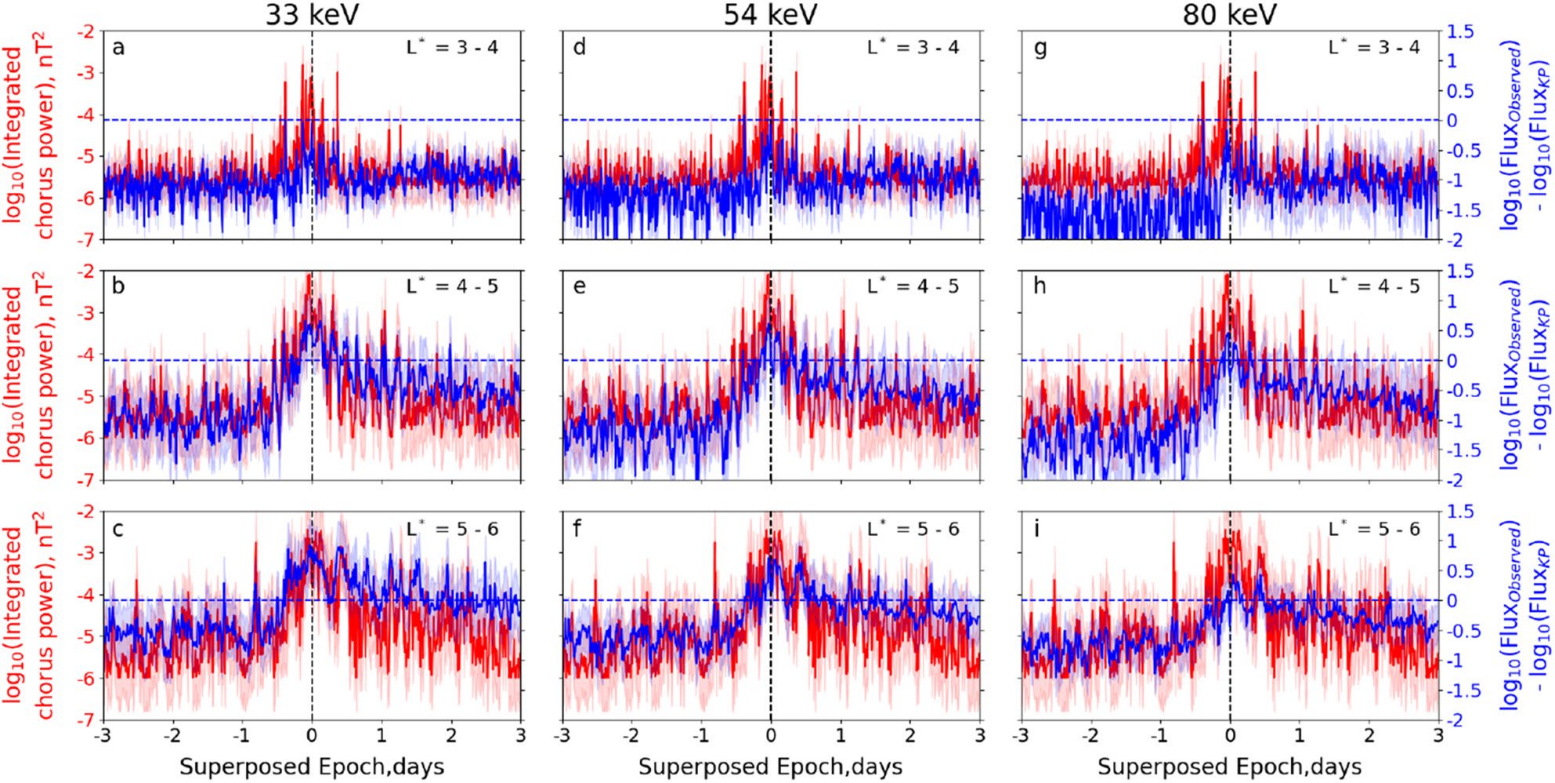
Superposed epoch analysis of integrated chorus wave power (0.1–0.8 f$_{ce}$; nT^2^; red curves) and difference of observed flux and calculated KP limiting flux (blue curves) in logarithmic scale as a function of superposed epoch (in days) at three different electron energy channels: (**a**–**c**) 33 keV, (**d**–**f**) 54 keV and (**g**–**i**) 80 keV, and three different L$^*$ ranges: (**a**, **d**, **e**) L$^*$ = 3–4, (**b**, **e**, **h**) L$^*$ = 4–5 and (**c**, **f**, **i**) L$^*$ = 5–6, between 0 to 12 MLT. See text for details.

First, we examine the statistical variation in time of integrated chorus wave power and the ratio of observed flux to calculated KP limited flux in three $L^*$ ranges. Figure 2 illustrates the variation of the integrated chorus wave power (red curves) and the ratio of the observed flux and calculated KP limited flux (blue curves) on a logarithmic scale as a function of superposed epoch (in days). Three energy channels (33 keV, 54 keV, and 80 keV) are shown in each column, and three L$^*$ ranges ($3 - 4$, $4 - 5$, and $5 - 6$) are shown in each row. The solid lines are the median values and the shaded regions are their standard deviations. In each panel, the vertical black dashed line marks the zero epoch and the horizontal blue dashed line indicates where the observed flux is equal to the KP limit. Several important features can be noted from Fig. 2:

1. In the region $3< L^* < 4$ during the storm main phase (near epoch day 0), the observed flux reaches close to the KP limited flux, within an uncertainty factor of 3 (panels a, d, g). Note that this is the same uncertainty as assumed by Kennel and Petschek^26^ in their original paper. The flux never exceeds the KP limit. The integrated chorus wave power exhibits a few intense noisy burst-like peaks ($P_{ch} \sim 10^{-3}$ nT$^2$) during the same time interval, which are well-correlated with the enhancements in electron flux.
2. In the region $4< L^* < 5$, and in most storms, the observed flux consistently exceeds the KP limited flux during the storm main phase (panels b, e, h). The flux at the lowest energy channel (33 keV) exhibits a higher value than the other two higher energy channels. Once the flux exceeds the KP limit, it is brought down below the limiting flux within $\sim$ 1 day, although the flux never decays to its pre-storm level during the 3 day period after the zero epoch. In this L$^*$-range, the chorus wave power increases by almost 3 orders of magnitude above the pre-storm level, between epoch day $\sim -1$ and epoch day 0, where it reaches its maximum (with median $P_{ch} \sim 10^{-2}$ nT^2^). Subsequently, it takes $\sim$ 1 day to return back to its pre-storm level. There also appears to be a strong correlation between the chorus wave power and the fluxes during the recovery phase at shorter time scales, on the order of hours, across the entire ensemble of events. Such correlation is visible not only in the median values but also in the standard deviations.
3. In the region $5< L^* < 6$ the observed flux of 33 keV electrons exceeds the KP limited flux during the storm main phase and remains above the limit for almost 3 days after zero epoch (panel c). In comparison, the fluxes of 54 keV and 80 keV electrons only exceed the KP limited flux briefly during the storm main phase, followed by a gradual reduction to the KP limit during the storm recovery phase (panels f and i). The integrated chorus wave power also shows significant increase during the storm main phase, the median $P_{ch}$ reaching $\sim 10^{-2}$ nT$^2$ at epoch day 0. Subsequently, the wave power reduces to the pre-storm level after $\sim$ 1 epoch day. Similar to L$^*$ range $4 - 5$, $P_{ch}$ exhibits significant fluctuations during the entire recovery phase. There is also a good correlation between the chorus wave power and the fluxes.

Overall, Fig. 2 therefore shows that the most intense chorus waves are generated only when the observed flux exceeds the KP limit (as for $4< L^* < 6$), or is within a certain uncertainty factor ($\sim 3$) of the limit (as for $3< L^* < 4$). It should be noted that the relationship between the generation of intense chorus waves and electron flux exceeding the KP limit is much stronger for $4< L^* < 6$ than for $3< L^* < 4$. Below, we focus our further analysis on the region $4< L^* < 6$ that covers the heart of the outer radiation belt.

**Figure 3:**
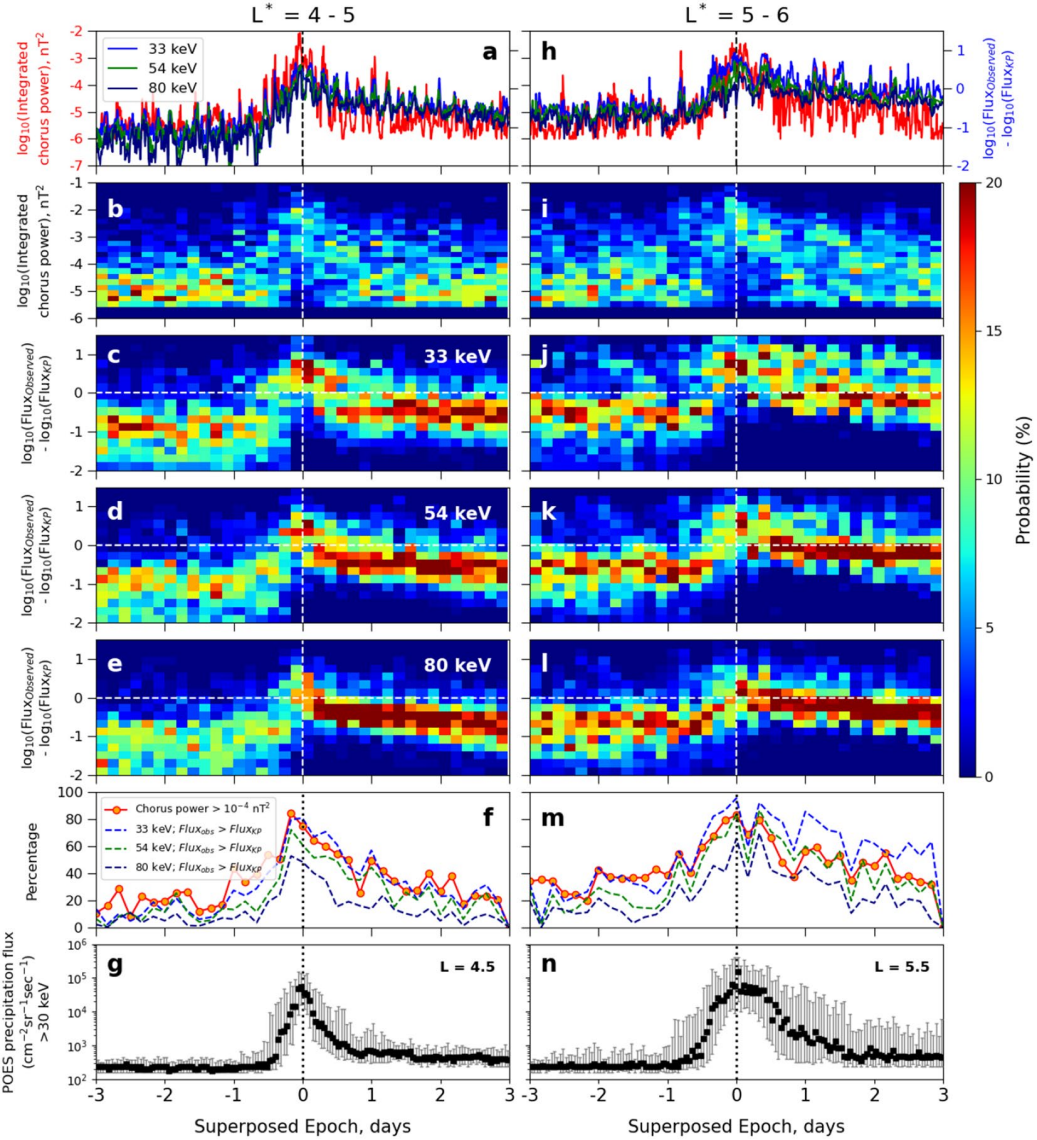
Median (**a**, **h**) integrated chorus wave power (nT^2^; red) and difference of observed and calculated KP limiting flux for 33 keV (blue), 54 keV (green) and 80 keV (navy) electrons; probability distribution function (PDF) of (**b**, **i**) integrated chorus wave power and difference of observed and KP limiting flux for (**c**, **j**) 33 keV, (**d**, **k**) 54 keV and (**e**, **l**) 80 keV electrons in logarithmic scale; (**f**, **m**) percentage of finding integrated chorus wave power $> 10^{-4}$ nT^2^ and observed flux greater than KP limiting flux for 33 keV (blue), 54 keV (green) and 80 keV (navy) electrons within the L$^*$ range 4–5 (left panel) and 5–6 (right panel); and precipitating flux as observed by POES for > 30 keV electrons at (**g**) L = 4.5 and (**n**) L = 5.5 as a function of superposed epoch (in days) between $0 - 12$ MLT. In each panel, the vertical dashed line marks the zero epoch and the horizontal dashed lines in panels (**c**–**e**) and (**j**–**l**) indicate the observed flux being equal to the KP limiting flux. The colorbar at the right denotes the PDF, so that the probability of finding events in each vertical slice adds up to 100%. In panels (**g**, **n**), the black scatter plot shows median electron flux and the error bars represent upper and lower quarterlies of the superposed epoch statistics.

To further emphasize the relationship between intense chorus waves and the flux of the source electron population, in Fig. 3 we present the median values (top row) and the probability distribution functions (PDFs) of both the integrated chorus wave power (second row) and the ratio of the observed and calculated KP limit for 33 keV, 54 keV and 80 keV energy electron (third to fifth rows). A logarithmic scale is used, and we consider the region with L* values 4–5 (left panel) and 5–6 (right panel), within 0–12 MLT. Figure 3 panels (a and h) contain some of the same data as the middle and bottom rows of Fig. 2, which we augment with probability distribution functions (PDFs) to provide further insight. To construct the PDFs, we have taken a 4 h time window and present normalized histograms of the log chorus wave power and log flux ratios (observed flux to KP limit) with vertical bin widths of 0.2, such that the probability of finding events in each given time slice adds up to 100%. Panels (f) and (m) show the percentage of events in each vertical slice where either $P_{ch}>10^{-4}$ nT^2^, or the electron flux in each of the three energy channels exceeds the relevant KP limit. The bottom panels (g and n) show the precipitation flux as observed by the Polar Operational Environmental Satellites (POES) for >30 keV electrons at two specific L shells within the corresponding L* range. For these panels, we considered the same set of 70 geomagnetic storms during the Van Allen Probe era, and used the 0° telescope to reveal the precipitation fluxes in the dawn sector (0 to 12 MLT). At these L-shells, 0° telescope measures only precipitating particles with equatorial pitch angle of ~1.5°.

First, we focus on the $4< L^* < 5$ region, as this is where the chorus waves are at their most intense (compare Fig. 3a with Fig. 3h). Before and after the storm main phase, between epoch days − 3 to − 1 and from epoch days 1–3, chorus waves are more likely to have $P_{ch}<10^{-4}$ nT^2^ (Fig. 3b). During the storm main phase, between epoch days − 1 to + 1, the probability of observing $P_{ch}>10^{-4}$ nT^2^ is significantly increased. As time progresses from epoch day -1 to epoch day 0, $P_{ch}$ increases dramatically so that at epoch day 0, almost all $P_{ch}$ is $> 10^{-4}$ nT^2^, before returning to nearly pre-storm levels at epoch day 1. From Fig. 3c–e, we can see that before epoch day $\sim -1$, the flux ratios are below the KP limit and the PDFs are wide. After epoch day $\sim -1$, the probability of finding the observed flux greater than the KP limit begins to increase for all energies. At epoch day 0, the probability is maximised at values above the KP limit. The most important difference before and after the storm main phase is that after epoch day 0, the PDFs of electron flux become significantly concentrated with very high probabilities for the observed flux being close to the KP limited flux. From panel (g), we can see that starting from epoch day -0.5, the precipitating flux of electrons having energies > 30 keV at L = 4.5 starts to increase, reaching a maximum at epoch day 0, after which it begins to decrease. After epoch day 1, the precipitating flux reduces back to its pre-storm level. This is in strong correlation with the variation of both the integrated chorus wave power (panel b) and the electron fluxes (panels c–e), and shows that when the observed fluxes of tens of keV electrons exceed the theoretically predicted KP limiting flux, intense chorus waves are generated that lead to the precipitation of electrons into the atmospheric loss cone, exactly as predicted by Kennel and Petschek in their 1966 paper^26^.

The observations presented in Fig. 3 panels (a-e) suggest that once the flux crosses the KP limit during the storm main phase, it is essentially capped at the limiting value in the storm recovery phase, and that the process causing the cap is associated with intense chorus activity. As discussed in the previous paragraph, panel g further supports this theory that it is the wave-particle interactions with the intense chorus waves that cause atmospheric precipitation of electrons, thereby limiting the radiation belt fluxes to the theoretically predicted limit. To understand this feature explicitly, we checked the percentage of finding chorus wave power $P_{ch}>10^{-4}$ nT^2^, and observed flux greater than the KP limit, which is presented in Fig. 3f. The value of $10^{-4}$ nT^2^ was chosen based on an examination of the superposed epoch response of the storms from Fig. 2. From this panel, we can see that the likelihood of finding $P_{ch} > 10^{-4}$ nT^2^ (red curve) increases during the storm main phase, with a maximum ($\sim 85\%$) at epoch day 0. After this time, the likelihood gradually decreases to pre-storm level. Interestingly, the likelihood of observed flux being greater than the KP limited flux (blue, green and navy dashed curves) exhibits almost identical behaviour for all the three energy channels. There seems to be a strong correlation between the chance of seeing flux values above the KP limit and the change of seeing intense chorus wave power, especially for $E=33$ keV. Overall, this is strongly supportive of the hypothesis that the enhancement of the absolute value of electron flux above a theoretically-derived limit during storm main phase is responsible for the generation of intense chorus wave power for $4< L^* < 5$.

In the region with L* values between 5 and 6 (Fig. 3, panels i - l), although the overall features of the flux PDFs remain the same as at L$^* = 4 - 5$, there are some notable differences. For the waves, Fig. 3 panel (i) demonstrates that before epoch day $\sim -1$, the chorus wave power is mostly below $10^{-4}$ nT^2^, after which it begins to increase and becomes maximum (wave power $\sim 10^{-2}$ nT^2^) at epoch day 0. However, after epoch day 0, differently to $4< L^* < 5$, $P_{ch}$ is more likely to remain high and doesn’t reduce to its pre-storm level over the following 3 days. The wave power also exhibits significant variation during the storm recovery phase (days 1–3), with a much wider distribution than before. For $5< L^* < 6$, Fig. 3j–l show that the observed flux is below the KP limit for all the three energy channels before epoch day $\sim -1$. For this higher $L^*$ range, the PDFs are not wide, rather the observed fluxes are more likely to be close to but below the KP limit. After epoch day $\sim -1$, the probability of finding observed flux exceeding the KP limit increases, and becomes maximum at epoch day 0. After epoch day 0, the observed fluxes of 54 keV and 80 keV electrons (Fig. 3 panels k and l) exhibit similar behaviour as in the L* range $4 - 5$, i.e., they are capped at the KP limit and the PDFs become significantly narrowed with values close to the KP limit. But for 33 keV electrons (Fig. 3j, after epoch day 0, the observed flux can exceed the KP limited flux for a longer period of time, and the PDF is more widely distributed in this energy channel. This feature can be seen more clearly in Fig. 3m. The probability of finding observed flux of 33 keV electrons greater than the KP limit (blue dashed curve) again increases during the storm main phase, being maximum at epoch day 0. After epoch day 0, although the percentage shows a decreasing trend, it still remains high and exhibits some recurrent crests and troughs. Interestingly, it is during these same crests that the high chorus wave powers (red curve) are observed in the storm recovery phase. This suggests that a strong correlation between chorus waves and the flux of 33 keV electrons exists despite the fluctuations and can be maintained outside of the storm main phase. Therefore whenever and wherever the flux of energetic electrons exceeds the KP limit, intense chorus waves are more likely to occur. The precipitating flux in this L* range (panel n) also exhibits a notable difference from that in the L* range 4–5 (panel g), although they are in good correlation with the trapped fluxes (panels j–l). Panel (n) shows that the precipitating flux at L = 5.5 starts to increase from epoch day − 1, becoming maximum at epoch day 0, after which it is more likely to remain high and doesn’t reduce to the pre-storm level before epoch day 2. This is in good agreement with the trapped 33 keV flux variations as observed by the Van Allen Probes (panel j). Even the storm to storm variability as can be seen in both the PDFs (panel j) and scatter plot (panel n) are well correlated which supports the fact it is the precipitation resulting from wave-particle interactions that maintains the trapped fluxes at the predicted KP limit.

**Figure 4:**
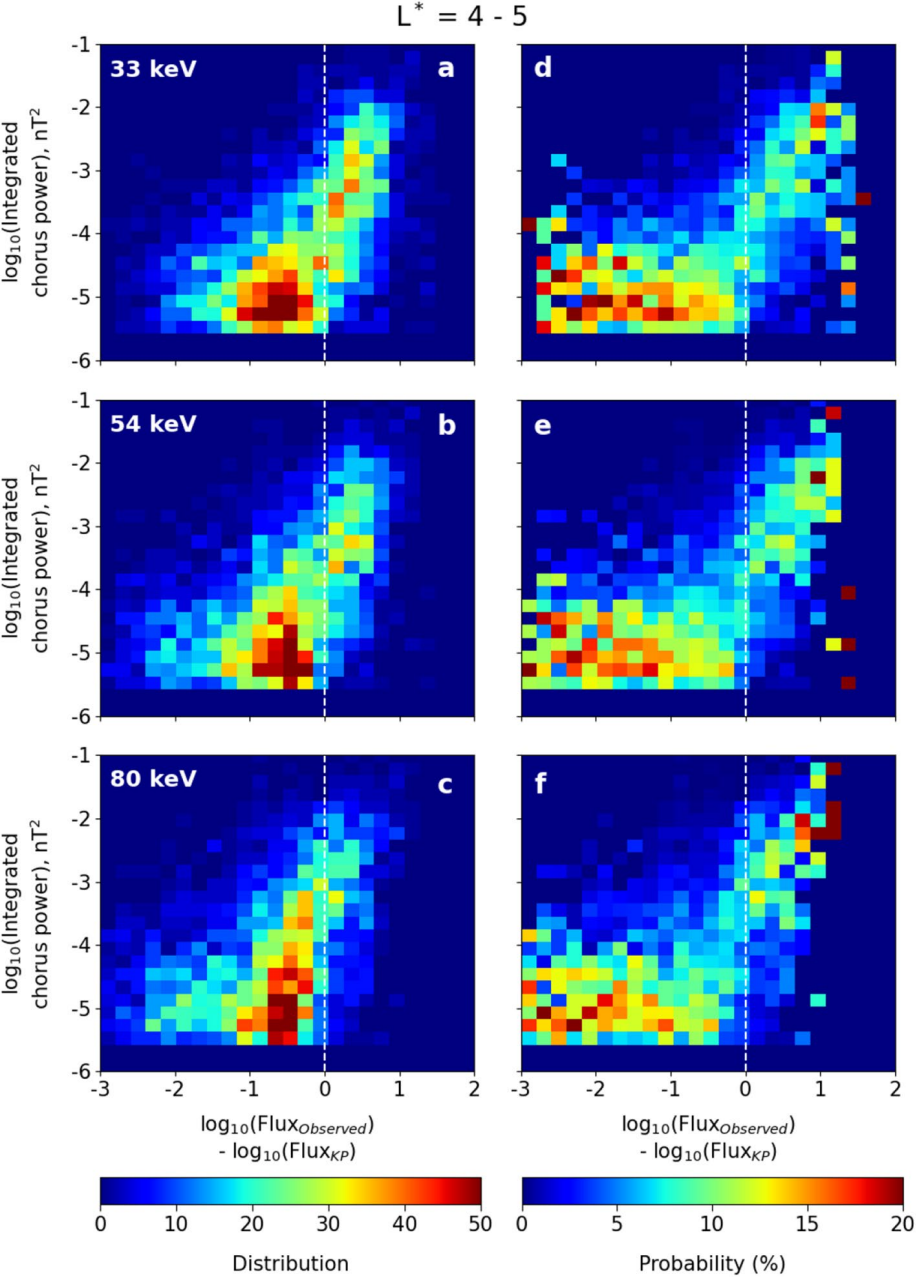
(**a**–**c**) Two dimensional histograms and (**d**–**f**) probability distribution functions (PDFs) of integrated chorus wave power against the logarithm of the ratio of the observed flux to the calculated KP limit. The plots are in log-log space for (**a**, **d**) 33 keV, (**b**, **e**) 54 keV and (**c**, **f**) 80 keV energy electrons within the L* range $4 - 5$. The colorbars at the bottom denote the distribution (number) of data points (left column) and probability (right column) of finding a given chorus wave power at a given flux ratio, using bins having bin width of 0.2 × 0.2 in log-log space. The vertical dashed line in each panel indicates that the observed flux is equal to the KP limit.

**Figure 5:**
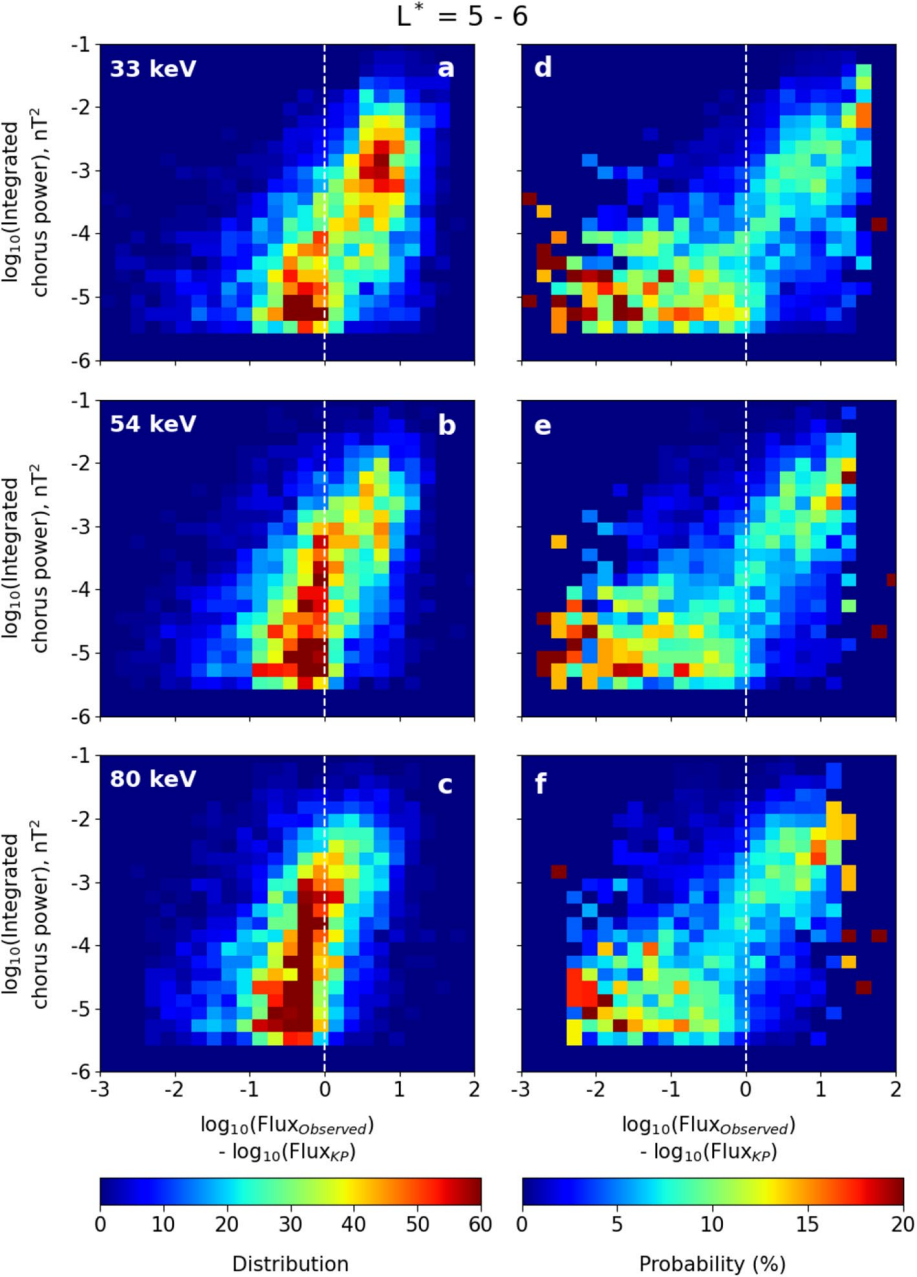
Same as in Fig. 4, but in the L* range $5 - 6$.

Our final statistical test is to remove the temporal information about the storm evolution, and study the probability that high values of electron flux results in intense chorus waves. We provide two-dimensional histograms and PDFs of integrated chorus wave power and the ratio of the observed flux to the KP limit in log-log space for three energy channels (33 keV, 54 keV and 80 keV) in the L* range $4 - 5$ (Fig. 4) and $5 - 6$ (Fig. 5). To construct the 2D histograms and normalized PDFs, we have taken bins with a bin width 0.2 × 0.2 in this log-log space. In both Figs. 4 and 5, panels (a–c) show the distribution of the number of observations in that 2D bin across the 70 chosen storms, while panels (d–f) show normalised PDFs. Here the percentage of chorus power at different intensities is plotted as a function of the ratio of the observed flux to the KP limit.

The two-dimensional histograms and PDFs in Figs. 4 and 5 demonstrate that there is very different wave behaviour when the electron flux is less than the KP limit (to the left of the dashed white line) than when the flux is above the KP limit (to the right of the dashed white line). The PDFs panels (d–f) in particular show that when the flux is below the KP limit, the waves are most likely to have $P_{ch}<10^{-4}$ nT^2^, with a distribution that does not depend upon the value of the flux. For electron fluxes above the KP limit, the probability distribution function of the waves is shifted to dramatically higher values, and exhibits a strong dependency on how much the electron flux exceeds the KP limit. The peak of the PDFs in panels (d–f) jump by orders of magnitude as the KP limit (the white dashed line) is crossed. Above the KP limit, there is some evidence of a power law relationship between the chorus power and electron flux, where the PDFs can be fitted by a straight line with slope $\sim 2$.

**Figure 6:**
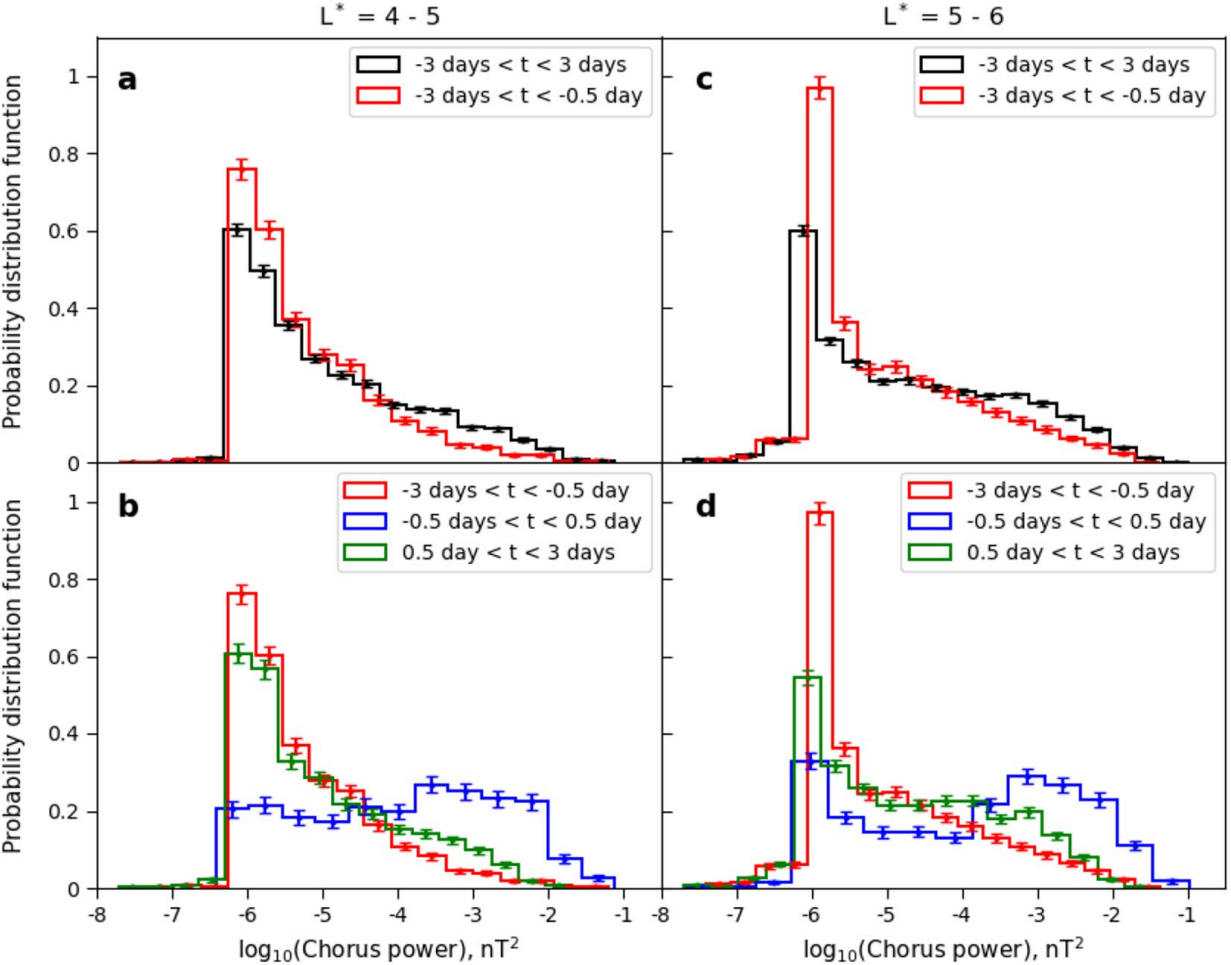
Normalised chorus wave power occurrence distribution in different time intervals in the L* range 4–5 (**a**,** b**) and 5–6 (**c**,** d**). Chorus wave power in logarithmic scale is plotted along the x axis and the normalised occurrence distribution is plotted along the y axis.

Once the temporal behaviour of the storm is removed, there is very little difference in wave and flux relationships between $4< L^* < 5$ and $5< L^* < 6$, indicating that the underlying physical process is the same (compare Figs. 4 and 5). There are clearly two very distinct populations of chorus power, separated by the proximity of the electron flux to the KP limit. When the flux is significantly below the KP limit, the chorus waves have an occurrence distribution which is variable but not strongly controlled by the magnitude of the flux. This is likely associated with an ambient level of chorus wave power that exists due to a temperature anisotropy of the plasma^31^. However, when electron fluxes exceed the KP limit the chorus wave power occurrence distribution comprises almost exclusively of just the most intense waves. In Fig. 6 we show that this population of very high power chorus waves is indeed distinct from the lower power background distribution, and that this transition occurs once the fluxes reach the KP limit. Figure 6a and c compare the distribution of $P_{ch}$ over the entire storm centred around epoch day 0 ($\pm 3$ days; black line) with the distribution of $P_{ch}$ for the pre-storm phase (from − 3 days to − 0.5 days; red line). In the pre-storm phase, there is significantly more likelihood of low power chorus ($P_{ch} \sim 10^{-6}$ nT^2^) and a big decrease in the likelihood of observing power in the $10^{-4}$ - $10^{-2}$ nT^2^ range. If we further investigate the storm progression by isolating pre-storm (red), main phase (blue) and recovery phase (green) in Fig. 6b and d, then the main phase chorus exhibits a very different occurrence distribution indicating the presence of an additional distribution of intense chorus waves that are not present at other times. Thus, Fig. 6 is testimony to the fact that the occurrence of chorus waves with extreme wave power are preferentially generated during periods when the electron flux is very likely to exceed the KP limit.

## Discussion and conclusions

The statistical observations presented here demonstrate that whenever and wherever the flux exceeds the KP limit during geomagnetic storms, intense chorus waves are typically generated. Kennel and Petschek^26^ suggested that the flux of stably trapped electrons in a magnetosphere would be capped by the action of intense chorus waves that rapidly grow to large amplitudes and scatter electrons into the loss cone to precipitate into the upper atmosphere. Importantly, this study reveals the existence of two key regimes for chorus waves in the outer radiation belt. The first, and far more common regime is that where the flux of 10–100 keV electrons falls below the KP limit. In this study, this regime covers the three days prior to a geomagnetic storm, and most of the recovery period after the main phase. It is likely that most intervals of time, outside the short periods characterized by geomagnetic storms have levels of 10–100 keV electron flux that fall below the KP limit. At these times, chorus wave power rarely exceeds $10^{-4}$ nT^2^. The generation mechanism for whistler mode waves under typical conditions has been previously identified as typically being the result of perpendicular temperature anisotropy^31^. Wave amplitudes may be enhanced by increasing partial number density of the warm plasma component^1^, and likely also by the strength of instability arising from pitch angle anisotropy, but our statistical analysis demonstrates that the amount of integrated chorus power has little relationship with the value of the flux in separate energy channels in this first regime.

The second regime is much more rare, and occurs when the electron flux exceeds the KP limit. In this case, Kennel and Petschek^26^ suggested that the source of the anisotropy necessary for the wave instability is unimportant; growth rates of chorus will become high because they are dependent upon the absolute value of the flux, and that when this becomes unusually high, so do the wave growth rates. Particle scattering rates depend on the wave power, and the wave power depends upon how quickly the waves can grow before they propagate away from the source region^39,40^. Above the KP limit, the plasma is predicted to become strongly unstable to chorus wave growth, where an external source, such as radial diffusion, or substorm electron injection maintains a flux level above the KP limit. Intense chorus waves are then generated that rapidly scatter electrons into the loss cone. The “excess plasma” above the KP limit is lost to the atmosphere, and the waves propagate away from the region, guided close to the magnetic field^40,41^. The quasi-steady balance of rapid wave growth, pitch angle scattering and precipitation loss is maintained as long as the flux levels remain above the KP limit. Crucially, in this state, the wave power depends upon how much the flux exceeds the KP limit, as our observations show. The existence of the two regimes, and the dependence of wave power on how much the electron flux exceeds the KP limit, provide direct evidence that the flux-limiting process predicted by Kennel and Petschek over 50 years ago operates in the terrestrial magnetosphere during geomagnetic storms.

**Figure 7:**
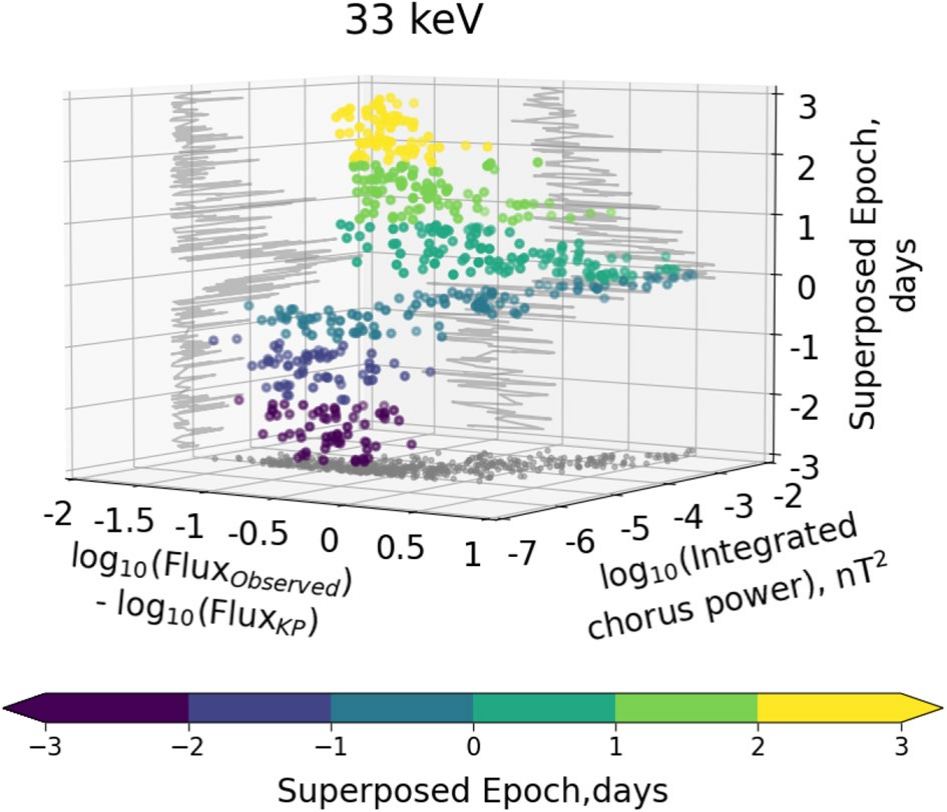
Scatter plot of the median integrated chorus wave power (in nT^2^) and ratio of observed and KP limited flux (log scale) of 33 keV electrons in the L* range 4–5, as a function of superposed epoch (in days) in a three dimensional space (dots show the data, with the colour scale indicating the superposed epoch time - right axis). Projections onto the respective two dimensional planes are plotted in gray. The colorbar denotes the superposed epoch in days.

To summarize the overall results more clearly, we further present scatter plot of the median of integrated chorus wave power (in nT^2^) and the median ratio of the observed flux to the KP limit as a function of superposed epoch (from 3 days before and after the epoch time zero at storm minimum Sym-H) in a three dimensional space with projections on the respective two dimensional planes, in the L* range $4 - 5$ (Fig.7). Figure 7 show clearly the relationship between the generation of intense chorus waves and the dynamics of tens of keV electron flux during the course of a storm. It is only during the storm main phase, and only once the electron flux exceeds the KP limit, that intense chorus waves are generated. In the pre-storm period, when fluxes are below the KP limit, the chorus wave power is much lower (of the order of 10$^{-6}$ to 10$^{-5}$ nT^2^) and represents a separate and distinct more ambient population. During the recovery phase, following a period of intense chorus wave generation once the fluxes exceed the KP limit during the main phase, the chorus wave power again returns to the separate and distinct lower power and more ambient population. To visualize the progression of both flux and integrated chorus wave power during the course of geomagnetic storms, we have provided a movie in the supplementary material (Fig. S2).

Very large amplitude whistler-mode waves have been previously observed across both the outer radiation belt^42–50^, and indeed across the closed magnetosphere region up to $L=10$^45^. Although these studies provide a large data set of high amplitude whistler mode waves from which their statistical spatial extent is well-known, the mechanism responsible for the generation of such large amplitude waves is not well understood. In our view, this subset of intense magnetospheric wave activity likely includes periods of flux limitation due to the process suggested by Kennel and Petschek. Future work will examine whether generation of such intense chorus waves can be explained by the KP process, or whether other generation mechanisms are also possible. The large-amplitude waves in this study are likely to be so large that the quasi-linear theory upon which the original Kennel–Petschek analysis rests is less applicable. However, the observations indicate that the general predictions of the flux-limiting process are observed in the magnetosphere, i.e. that above a particular threshold, the amount of flux at particular energies is related to the size of the whistler-mode waves. Future analysis should include nonlinear effects of large amplitude waves (e.g.,^51–57^) to derive the balanced equations that describe what happens to the wave-particle interaction once the threshold has been reached. The observational analysis here indicates that the value of the threshold as determined by quasi-linear theory is a reasonable approximation for conditions experienced in Earth’s inner magnetosphere.

Numerical models of the radiation belt based upon a Fokker–Planck description of wave particle interactions are extensively used worldwide for scientific analysis of prior geomagnetic events^58^, reanalysis of decades of historical data^59^ and in numerical space weather prediction (e.g., see^60^). To our knowledge, except a few studies (e.g.,^55,61^), none of these models specifically incorporate the diffusion models necessary to provide the rapid flux-limiting demonstrated in our observations. Future studies should also identify the impact of flux-limiting intense chorus waves on electrons at higher energies (e.g. $>1$ MeV) in addition to creating descriptions appropriate for their incorporation into numerical radiation belt models. Overall, our work shows that intense chorus waves are excited as part of the natural self-limiting of the flux of electrons in the radiation belts, exactly as first predicted by Kennel and Petschek^26^ more than 50 years ago.

## Methods

### Calculation of difference between observed flux and KP limiting flux

In this study, we have used observations of electron fluxes from the Magnetic Electron Ion Spectrometer (MagEIS) instrument on board the Van Allen Probe-A spacecraft. The MagEIS instrument, which is part of the Energetic Particle, Composition, and Thermal Plasma Suite (ECT^30^), provides 11 second resolution of spin averaged (Level 2) and pitch angle resolved (Level 3) electron flux measurements at 25 electron energy channels. For this study, we considered the Level 3 electron flux data measured at $90^\circ$ pitch angle at the three lowermost energy channels, viz., 33 keV, 54 keV and 80 keV, during 70 geomagnetic storms in the Van Allen Probe era ($2012 - 2019$). The storms are selected with the criterion that each of them are isolated events with minimum SYM-H index less than $-50$ nT. The details of the storms can be found in Olifer et al.^27^.

We calculate the KP limited flux using the methodology introduced by Mauk and Fox^28^. It was also used in the original Olifer et al.^27^ paper to analyze the 70 isolated geomagnetic storms with SYM-H $\le -$50 nT during the Van Allen Probe era—the same set of storms as we use in this study. The algorithm for the calculation of the KP limit by Mauk and Fox^28^ formulates the problem in terms of the differential flux following the earlier studies by Schulz and Davidson^62^ and incorporates the relativistic corrections from Summers et al.^63–65^. Similarly to the original KP paper, Mauk and Fox^28^ state that the KP limit for electrons is defined as the electron flux level at which chorus wave generation is sustained by the pitch angle anisotropy and which balances the losses due to wave partial reflection at the ionosphere. The KP limit is defined by balancing partial reflection from the ionosphere and additional growth of the reflected wave in the equatorial region. This leads to a condition $G \cdot R=1$, where *G* is a net gain of whistler wave amplitudes along the field line and *R* is the ionospheric reflection coefficient. Mauk and Fox^28^ use this condition, as well as expressions for the *e*-folding temporal growth rate introduced by Xiao et al.^66^, to calculate a KP limit based on the observed electron flux spectrum. We refer the reader to the original Mauk and Fox^28^ paper for a more detailed description of the approach used to estimate the differential flux at the KP limit.

For the purpose of this study, we use a similar approach for calculating superposed epoch electron flux with respect to the resulting KP limit as was introduced by Olifer et al.^27^. Both the observed flux and KP limit are binned in 50 L* bins between L* of 1.0 and 7.5 and in 120 superposed epoch bins between $-3$ and 3 superposed epoch days for each storm, with zero epoch denoting the time of minimum SYM-H in every event. The binned electron fluxes and their ratios for different energy channels in each selected storm are then used to determine the median and standard deviation in each of the bins.

### Calculation of integrated chorus wave power

To investigate chorus wave activity, we have taken 6 second resolution wave magnetic field measurements provided over 65 logarithmically spaced frequency intervals between $\sim 1$ Hz to $\sim 12$ kHz from the Electric and Magnetic Field Instrument Suite and Integrated Science (EMFISIS;^32^) on board the Van Allen Probe-A spacecraft. To ensure that the observed waves are indeed chorus waves, we looked at the background plasma density measured by EMFISIS instrument onboard the Van Allen Probes, and selected waves when the spacecraft was outside the plasmasphere. Then, from the wave magnetic field measurements, we calculated 5 min averaged integrated chorus wave power in the frequency range 0.1–0.8 f$_{ce}$, where f$_{ce}$ is the equatorial electron gyrofrequency. We used the same 50 L* bins and 120 superposed epoch bins, as used for the fluxes. Similarly, the binned integrated chorus wave power in each selected storm are then used to calculate the median and standard deviation in each bin.

## Supplementary Information

Supplementary Information.

## Data Availability

The data sets used in this study are publicly available. The interplanetary parameters and geomagnetic indices are obtained from the website https://cdaweb.gsfc.nasa.gov/cgi-bin/eval2.cgi. The Van Allen Probe data used in this study are available at the websites http://emfisis.physics.uiowa.edu/Flight/ for EMFISIS, and http://www.rbsp-ect.lanl.gov/data_pub/ for ECT. The POES data used in this study can be found at https://www.ngdc.noaa.gov/stp/satellite/poes/dataaccess.html.
