# Supplementary material for: Intense chorus waves are the cause of flux-limiting in the heart of the outer radiation belt
Source: Sci Rep. 2022 Dec 15;12:21717. doi: 10.1038/s41598-022-26189-9 (PMC9755534; doi:10.1038/s41598-022-26189-9)
Supplement: Supplementary file 1 — Supplementary Information. [file 41598_2022_26189_MOESM1_ESM.pdf]

Supplementary Material for "*Intense chorus waves are the cause of flux-limiting in the heart of the outer radiation belt*"

S. Chakraborty[1,*], I. R. Mann[2,1], C. E. J. Watt[1], I. J. Rae[1], L. Olifer[2], L. G. Ozeke[2], J. K. Sandhu[1], B. H. Mauk[3], and H. Spence[7]

[1]Department of Mathematics, Physics and Electrical Engineering, Northumbria University, Newcastle upon Tyne, UK
[2]Department of Physics, University of Alberta, Edmonton, AB, Canada
[3]Applied Physics Laboratory, Johns Hopkins University, Laurel, MD, USA
[7]Institute for the Study of Earth, Oceans, and Space, University of New Hampshire, Durham, NH, USA
[*]Corresponding author: S. Chakraborty, suman.chakrabarty37@gmail.com

1 Supplementary Information

Figure S1 provides the statistical variation in time of integrated chorus wave power and the ratio of observed flux to calculated KP limited flux in three L^* ranges within the MLT range 12 - 24. The plots in each panel are in the same manner as Figure 2 in the main text. Figure clearly shows that the variation of integrated chorus wave power in noon to midnight MLT sector is less intense compared to the midnight to noon MLT sector.

2 Supplementary Figures

Figure S2 provides a rotating movie of the scatter plot of the points that comprise Figures 3, 4 and 5. Figure S2 shows the storm time evolution of the 33 keV electron flux and integrated chorus wave power in the L* range 4 - 5 and MLT range 0 - 12 as a function of superposed epoch (in days), represented by the colorbar at the bottom.

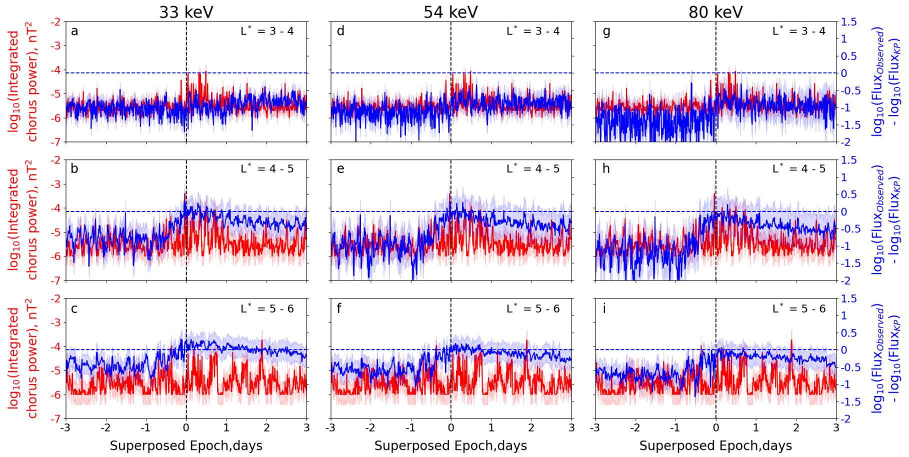

Figure S1 : Superposed epoch analysis of integrated chorus wave power (0.1 - 0.8 f_{ce}; nT^2; red curves) and ratio of observed flux to calculated KP limited flux (blue curves) in logarithmic scale as a function of superposed epoch (in days) at three different electron energy channels: (a - c) 33 keV, (d - f) 54 keV and (g - i) 80 keV, and three different L^* ranges: (a, d, e) $L^* = 3$ - 4, (b, e, h) L^* = 4 -5 and (c, f, i) $L^* = 5$ - 6, between 12 to 24 MLT.

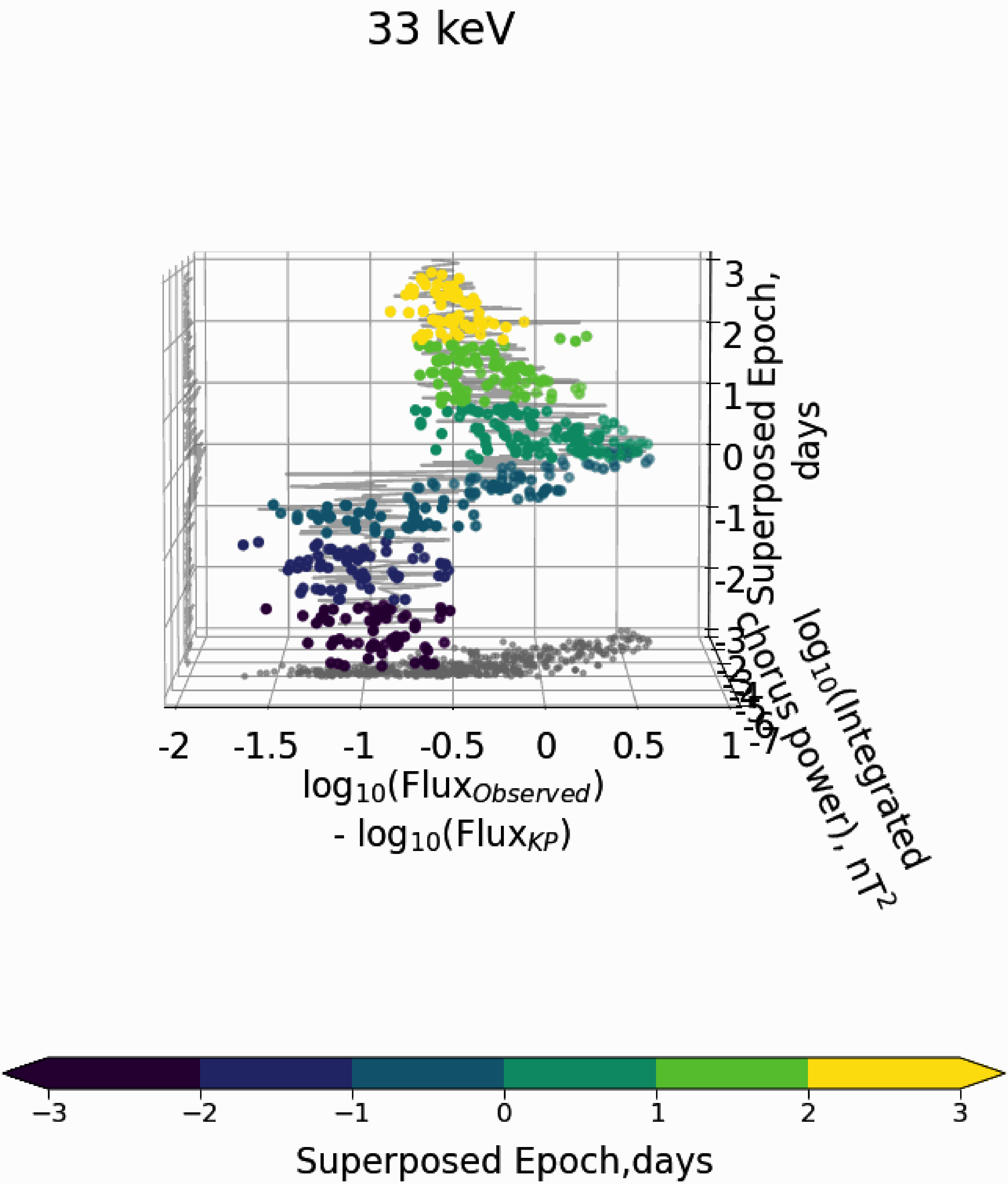

33 keV
Superposed Epoch, days
log10(Flux_Observed) - log10(Flux_KP)
log10(Integrated chorus power), nT^2
-2 -1.5 -1 -0.5 0 0.5 1
-3 -2 -1 0 1 2 3
Superposed Epoch,days
